# Design of the PROCON trial: a prospective, randomized multi – center study comparing cervical anterior discectomy without fusion, with fusion or with arthroplasty

**DOI:** 10.1186/1471-2474-7-85

**Published:** 2006-11-10

**Authors:** Ronald HMA Bartels, Roland Donk, Gert Jan van der Wilt, J André Grotenhuis, Dick Venderink

**Affiliations:** 1 Department of Neurosurgery, Radboud University Nijmegen Medical Centre, NL 6500 HB Nijmegen, The Netherlands; 2 Department of Orthopedic Surgery, Canisius Wilhelmina Hospital, NL 6532 SZ Nijmegen, The Netherlands; 3 Medical Technology Assessment, Radboud University Nijmegen Medical Centre, NL 6500 HB Nijmegen, The Netherlands; 4 Canisius Wilhelmina Hospital, Department of Radiology, NL 6532 SZ Nijmegen, The Netherlands

## Abstract

**Background:**

PROCON was designed to assess the clinical outcome, development of adjacent disc disease and costs of cervical anterior discectomy without fusion, with fusion using a stand alone cage and implantation of a Bryan's disc prosthesis. Description of rationale and design of PROCON trial and discussion of its strengths and limitations.

**Methods/Design:**

Since proof justifying the use of implants or arthroplasty after cervical anterior discectomy is lacking, PROCON was designed. PROCON is a multicenter, randomized controlled trial comparing cervical anterior discectomy without fusion, with fusion with a stand alone cage or with implantation of a disc. The study population will be enrolled from patients with a single level cervical disc disease without myelopathic signs. Each treatment arm will need 90 patients. The patients will be followed for a minimum of five years, with visits scheduled at 6 weeks, 3 months, 12 months, and then yearly. At one year postoperatively, clinical outcome and self reported outcomes will be evaluated. At five years, the development of adjacent disc disease will be investigated.

**Discussion:**

The results of this study will contribute to the discussion whether additional fusion or arthroplasty is needed and cost effective.

**Trial registration:**

Current Controlled Trials ISRCTN41681847

## Background

Since the first description of the cervical anterior discectomy with fusion by Cloward and Smith and Robinson in 1958 respectively in 1955[1,2], and the cervical anterior discectomy without fusion in 1960 by Hirsch[3] a debate is started which of both methods is the best. While this discussion is still not closed[4], the advent of the cervical disc prosthesis has contributed to extra confusion. Instead of two possibilities, nowadays three possible treatments concur with each other: cervical anterior discectomy without implantation of any structure (CAD), cervical anterior discectomy with fusion (CADF), and finally, cervical discectomy with implantation of a disc prosthesis (CADP).

Numerous clinical studies have been published. Several prospective, randomized trials have been reported [5-10]. However, methodological flaws as non homogenous patient population, undefined randomization process, small sample sizes, unclear outcome measurements and substantial loss of patients for follow – up, preclude definite conclusions regarding the efficacy of CAD versus CADF.

Recently, the results of a prospectively followed cohort have been published. They clearly showed that a cervical disc prosthesis is a safe devices[11]. However, one should bear in mind that the follow – up is short.

Several randomized controlled studies comparing arthroplasty and fusion with a plate have been reported with short follow – up or are still ongoing. CADF with a plate is defined as the gold standard[12,13]. This is very odd, since evidence has never been provided that cervical discectomy with fusion is better than without[4]. The use of a plate is even debatable[14].

Since the costs of implants are enormous, sound clinical evidence is needed to justify their use. Therefore, a prospective, randomized trial was developed comparing CAD, CADF using a cage, and CADP. Due to the experience of the principal investigators, the Bryan's disc prosthesis was chosen. The design of this trial is described and some of its strengths and limitations discussed. It is called the PROCON trial referring to the pro's and con's that are obvious present for each form of treatment.

## Methods/Design

### Study aims

PROCON has three aims:

1. to conduct a multicentre, randomized controlled trial comparing the clinical outcome of the different surgical options: CAD, CADF using a cage and, finally, CADP with implantation of a Bryan's disc prosthesis with a repeated longitudinal measurement up to 12 months.

2. to define differences in disc degeneration of the adjacent discs between the three surgical options. For this purpose, the patients will be followed for 60 months. All intercurrent treatments for cervical disc disease are recorded. A magnetic resonance imaging (MRI) scan will be performed 60 months after surgery.

3. to estimate the cost-effectiveness of the three surgical options.

### Study sites

The clinical centers that are planned to recruit patients into PROCON are located in the Netherlands: University Medical Center St. Radboud, Nijmegen; Canisius Wilhelmina Hospital, Nijmegen; St. Elisabeth Hospital, Tilburg, and finally, De Haaglande Hospital in The Hague.

### Study population

To obtain a homogenous patient population the following in – and exclusion criteria will be employed.

#### Inclusion criteria

All adult patients aged between 18 and 55 years with monoradicular signs and symptoms in the arm due to a herniated cervical intervertebral disc and/or an osteophyt at MRI are eligible for PROCON. The radiological findings should be in accordance with the clinical presentation. Furthermore, at the preoperative dynamic lateral X – ray the involved level should not have been fused. In – and exclusion criteria are represented in Table 1.

### Recruitment and Enrollment

Participating physicians at each site identify possible candidates. After their eligibility is controlled by one of the two principal investigators, the patients are informed about the PROCON trial. Seven days later, the patients are contacted again and informed consent is obtained from those willing to participate. Data regarding demographic characteristics, medical history and comorbidity, signs and symptoms, and baseline measurements for all outcomes are obtained through patient interview, patient self-administered survey, and physician survey.

### Randomization

For randomisation, the closed envelope method is used. As soon as informed consent is obtained, one of the treatment options is assigned to the patient. Prior to surgery, the patient is informed about the chosen option. Patients who do not choose for participation, are offered one of the surgical options that are currently under investigation. However, they are not followed in an observational cohort study.

### Study interventions

Three techniques are subject of study. A standard anterior cervical discectomy with the aid of a microscope is used in all cases. Whether the approach is from the left or right side is up to the preference of the surgeon. In case of CAD, the wound is closed after adequate decompression of the neural elements. However, if a fusion is chosen a cage (cervical I/F, Depuy Acromed, Johnson and Johnson, Amersfoort, The Netherlands) filled with bone substitute is placed within the intervertebral space. Although a needle technique for obtaining bone from the iliac crest with minimal pain has been described[15], the cage is filled with a commercially available bone substitute (beta – tricalcium phosphate) to prevent the pain from the iliac crest. The preparation for implantation of the Bryan's disc prosthesis (Sofamor Danek, Medtronic, Kerkrade, The Netherlands) is done before or after the standard cervical discectomy. Of course, the implantation of the prosthesis itself follows the discectomy. To prevent calcification along the prosthesis, the patients are prescribed meloxicam 15 mg daily for 14 days. The patients within the other two groups are prohibited to take non – steroidal – anti – inflammatory drugs (NSAIDs) for 14 days postoperatively.

All patients are encouraged to mobilize as soon as possible. A collar is never prescribed.

### Follow – up

Follow-up data are gathered at 6 weeks, 3 months and 12 months. Until 12 months the clinical assessment is done by an independent neurologist. Thereafter, the treating surgeon will coordinate follow – up and clinically assess the patient. At 12 months a computed tomography (CT) is made to evaluate fusion. In the prosthesis group, paravertebral calcifications within the long colli muscles can be ruled out. From one year until five years postoperatively, the patient is seen annually. At each visit plain X rays with flexion and extension of the cervical spine are made. Questionnaires are also filled out. Sixty months postoperatively, a MRI study is performed. Sagittal T_1_, T_2 _and proton density images will be obtained to obtain information about the adjacent disc.

### Outcomes

The primary outcome measure is the clinical and health-related quality of life after one year postoperatively as measured by both generic and disease specific instruments. Secondary endpoints include work status, fusion rate after 1 year, the development of a cervical kyphotic deformity at 1 and 5 years, and the incidence of adjacent disc disease at 5 years postoperatively.

### Primary outcomes

SF – 36 Health Status Questionnaire is a widely-used generic health status. This instrument consists of eight subscales and two summary scales. On each scale higher scores indicate better outcomes. Scores can be compared with published age – and sex – matched general population or disease-specific norms[16].

The McGill Pain score consists of four parts: 1) a list of words to describe the quality and intensity of the pain, 2) questions about the effects of the pain on daily life, 3) visual analogue scales, and 4) questions about the distribution and course of the pain. The McGill pain score is highly effective to measure the effects of a treatment on pain[17]. The Dutch version of this score is called the MPQ – DLV: McGill pain questionnaire – Dutch language version[17,18].

#### Neck Disability Index (NDI)

The NDI is a validated 10 – item questionnaire, that measures activity limitations due to neck pain. It is a self – reported instrument for the assessment of ADL, and it is a revised form of the Oswestry Low Back Pain Index[19,20].

#### Work Limitations Questionnaire (WLQ)

The WLQ is a 25 – item questionnaire developed to measure health – related decrements in ability to perform job roles among employed individuals. Patients themselves fill in the questionnaire. The WLQ scale scores have been proven to be sensitive to changes in health status over time [21].

### Secondary outcomes

The impact of surgery on working status is evaluated by calculating the duration before the patients fully resume normal activities. We will also document whether the patients are capable returning to their original jobs or if they have to change their work. The work limitations questionnaire is used to obtain a score that reflects the amount of discomfort the patient have performing their job. The pre-intervention scores are the baseline for follow – up. The mean differences are compared between the treatment arms.

Radiologic examinations are performed preoperatively and at each follow – up visit. At one year, a radiologist evaluates whether there is fusion on CT and plain X-Ray. The angle between the adjacent vertebrae is measured when the patient is holding the neck in neutral position while sitting. The angle between the endplate of the second cervical vertebral body and the upper endplate of the seventh cervical vertebra is also determined. Furthermore, this is also done when the patient maximally flexes or extends the neck. At five years postoperatively, the same evaluation is done. The development of anterior osteophytes of decrease of disc height of the adjacent levels is noted. Changes in time are determined within each treatment arm. The mean values are also compared between each treatment arm at 1 year and 5 year follow – up. Finally, at five years follow – up a MRI is made to evaluate whether a change of the quality of the intervertebral discs of the adjacent level can be shown.

### Cost – effectiveness

The aim of cost – effectiveness analysis is to reveal how differences in clinical outcome between the three surgical techniques relate to differences in their resource requirements. To this end, volumes of major cost drivers such as hospitalisation, medication use, out – patients consultations etcetera will be registered for each individual. For cost prices, national guidelines will be used. If differences are observed between the groups in resumption of work, costs of lost productivity will be estimated using the friction – cost method. Incremental cost – effectiveness ratios will be calculated: uncertainty of these estimates will be determined using bootstrap techniques[22].

### Monitored events

Monitored events are the death of a patient, withdrawal from the study, lost to follow – up, and cross – over from their randomly assigned treatment group. These events are registered within the case record form. The circumstances of the events are investigated and also noted. In case of death of the patient, a search for a relationship with the instituted treatment is started. Throughout the study, all medical complications and intervening treatments concerning the cervical spine are registered within the CRF at the usual follow – up visits or when the appropriate information reaches the treating surgeon.

### Protocol violations

Any of the following will be considered as a deviation from the protocol: randomization of an ineligible patient, enrollment of a patient that is already participating in an another study, enrollment of an PROCON participant in another study, a patient receiving the wrong treatment, loss of radiology or any other data, and informed consent violations. Violations are reviewed biweekly and reported to the independent study supervisor.

### Statistical Analysis

Our primary analysis is based on the intention to treat principle.

The primary study endpoints will be measured as changes from pre-intervention baseline scores. The mean scores of the three treatment arms will be compared at each follow-up time.

Sample size calculations were based on the outcome reported in literature. An excellent outcome (no complaints at all) is generally achieved in 60% of the patients, that underwent CAD or CADF. An increase of 20% was found acceptable to justify the use of a prosthesis.

With a power of 80 % and a two – sided level of significance of 0.05, a chi-square analysis would need 81 patients per group. Assuming a loss to follow – up of 10 %, a total of 270 patients are needed.

No subgroup analyses are planned, nor any interim – analyses.

### Organization of the study

The coordinating center of the study is located at the Canisius Wilhelmina Hospital, Nijmegen, The Netherlands, and at the University Medical Center Nijmegen St. Radboud. Technically, four groups exist within the organization. The first one is the trial direction, consisting of the leading investigators who also enroll patients in the study. They coordinate activities and liaise with other participating studies. The second one is the group of independent neurologists and radiologists. They are not involved in the coordination of the study. The third group is the medical technology assessment. They are involved in the development of the study protocol. They also control the input of data and perform statistical analysis of the data. The fourth and last group, is the independent supervising physician. He is a neurologist without experience in spine pathology. He does not examine patients for the study. Violations against the protocol are reported to him. He is also the contact person for those patients that have complaints not related to their disease or treatment (e.g. about the doctor). Patients can also contact him if they have questions during the study, about the study or are in doubt whether or not to participate.

## Discussion

Despite the lack of evidence in favour of one of the possible procedures, during the last decade the number of fusions has increased dramatically in the United States, whereas the total number of cervical spine procedures remained relatively constant[23]. The costs of fusion are considerable. In the Netherlands, a plate costs about 500 euro's and a cage about 700 euro's. The price of a disc prosthesis is even higher, about 2500 euro's. In the United States, disc prostheses are booming business. It is estimated that the market could approach $1.7 billion a year by 2010[24].

Apart from the clinical effectiveness, it is obvious that studies are needed to justify the enormous costs of fusion or arthroplasty. Several studies are conducted comparing arthroplasty with fusion. The design of this study is unique, since it does add an extra treatment arm, cervical anterior discectomy without fusion.

Several problems may arise. Randomization may not be easily accepted by patients. However, from a previous randomized controlled trial comparing surgical techniques[25], we learned that correct description of possibilities and estimated outcomes patients are not reluctant to participate. Especially the fact that no proof exists for one or another treatment will increase acceptance.

Surgery may have a placebo effect[26]. Clinical objective outcome is measured, but also self reported outcomes. PROCON is not able to determine a possible placebo effect. Although it must be considered a limitation of the study, its clinical value should also be questioned.

External validity and generalizability is often discussed. Patient selection is restricted to one level cervical disease without myelopathy. Therefore, the results cannot be extrapolated to two or more level disease, nor to patients with myelopathic symptoms and signs. On the other hand, the restriction to one level disease does prevent the need for stratification and loss of power during statistical analysis.

Although surgical skill may differ, a microscopic cervical anterior discectomy is a rather standard and straightforward technique. The use of the same implants does not allow variation due to construct design. Therefore, we do not feel that variation in skill will seriously affect the outcome of the study.

The second part of PROCON is the radiological evaluation of the development of adjacent disc disease. The preintervention plain X Rays are used as baseline study. The development of osteophytes or the increase of existing osteophytes may be a measure for the progression of adjacent disc disease. At five years postoperatively, an MRI is made to judge the quality of the adjacent discs. The question raises whether this time is long enough. At five years, adjacent disc disease may not have been fully developed, and perhaps its prevalence is not high enough to be statistically significant. However, extension of follow up time would severely compromise the inclusion of patients, and would also induce a high number of loss to follow up.

Finally, PROCON does measure costs. It is questionable if these findings can be extrapolated to other countries. First, the fee for hospitals and doctors are different. Secondly and probably most importantly, the compensation for sick leave is very differently arranged compared to other countries. Patients feel less urge to resume their daily working activities[27]. Therefore, the external validity of results of the cost effectiveness or cost minimization study will be extremely low, although tendencies might be formulated.

## Conclusion

Cervical anterior discectomy is frequently performed. The need for a randomized controlled trial comparing the cervical discectomy without fusion, with fusion and the newer technique arthroplasty is obvious. The costs of implants are enormous, whereas their effectiveness has never been proved. The design of such a study, and some of its limitations are discussed.

## Authors' contributions

R. B: generating the idea, writing the manuscript, epidemiological background

R. D: generating the idea, writing the manuscript;

G.J.vdW: epidemiological background

J.A. G: revising manuscript

D. V: formulating radiological part of the study.

## Pre-publication history

The pre-publication history for this paper can be accessed here:



## Figures and Tables

**Table 1:** Inclusion – and exclusion criteria

**Inclusion**	**Exclusion**
Age: 18 – 55 years	Symptoms and/or signs of myelopathy
Cervical monoradicular symptoms	Previous cervical surgery
MRI: herniated cervical intervertebral disc and/or osteophyte in accordance with clinical symptoms and signs	Psychiatric or mental disease
Involved level not fused	Involvement of liability procedure
	Alcoholism (drinking more than 5 units)
	Insufficient of the Dutch language
	Participation in another study
	Two or more levels involved
